# Testing a counselling intervention in antenatal care for women experiencing partner violence: a study protocol for a randomized controlled trial in Johannesburg, South Africa

**DOI:** 10.1186/s12913-016-1872-x

**Published:** 2016-11-05

**Authors:** Christina Pallitto, Claudia García-Moreno, Heidi Stöeckl, Abigail Hatcher, Catherine MacPhail, Keneoue Mokoatle, Nataly Woollett

**Affiliations:** 1 Department of Reproductive Health and Research, World Health Organization, Avenue Appia 20, 1211 Geneva, Switzerland; 2 Gender Violence and Health Centre, London School of Hygiene and Tropical Medicine, 15-17 Tavistock Place, London, WC1H 9SE UK; 3 Wits Reproductive Health and HIV Institute, University of the Witwatersrand, 22 Esselen Street, Hillbrow, 2001 South Africa; 4 School of Health, University of New England, Armidale, 2351 NSW Australia

**Keywords:** Intimate partner violence, Antenatal care, Counselling, Randomized controlled trial

## Abstract

**Background:**

Intimate partner violence (IPV) during or before pregnancy is associated with many adverse health outcomes. Pregnancy-related complications or poor infant health outcomes can arise from direct trauma as well as physiological effects of stress, both of which impact maternal health and fetal growth and development. Antenatal care can be a key entry point within the health system for many women, particularly in low-resource settings. Interventions to identify violence during pregnancy and offer women support and counselling may reduce the occurrence of violence and mitigate its consequences.

**Methods:**

Following a formative research phase, a randomized controlled trial will be conducted to test a nurse-led empowerment counselling intervention, originally developed for high-income settings and adapted for urban South Africa. The primary outcome is reduction of partner violence, and secondary outcomes include improvement in women’s mental health, safety and self-efficacy. The study aims to recruit 504 pregnant women from three antenatal clinics in Johannesburg who will be randomized to the nurse-led empowerment arm (two 30-min counselling sessions) or enhanced control condition (a referral list) to determine whether participants in the intervention arm have better outcomes as compared to the those in the control arm.

**Discussion:**

This research will provide much needed evidence on whether a short counselling intervention delivered by nurses is efficacious and feasible in low resource settings that have high prevalence of IPV and HIV.

**Trial registration:**

The study was registered in the South African Clinical Trials Registry (DOH-27-0414-4720) on 11 August 2014 and in the ISRCTN Registry (ISRCTN35969343) on 23 May 2016).

## Background

Violence against pregnant women is a global health concern. Studies have documented prevalence rates of partner violence during pregnancy ranging from 4–57 % globally [1–3]. South African studies estimate that 25–35 % of pregnant women experience past 12 month exposure to physical or sexual violence from a partner [4–6].

Intimate partner violence (IPV) during or around the time of pregnancy has been associated with many adverse health outcomes for the pregnant woman and her baby due to direct trauma, as well as physiological effects of stress from current or past violence that can impact fetal growth and development. Previous research has found that women experiencing violence during pregnancy had higher rates of intrauterine growth retardation and preterm labour [7–9], higher rates of miscarriage [10, 11], antepartum haemorrhage [12] and high blood pressure [13]. Violence during pregnancy has also been associated with maternal mortality [14] and perinatal, infant and child mortality [15, 16]. A recent systematic review and meta-analysis found that women reporting IPV in pregnancy were more likely to have a low birth weight infant and experience pre-term birth [17]. In addition, intimate partner violence has been associated with increased rates of HIV infection and HIV risk behaviour [18]. The maternal, infant, and reproductive health risks from intimate partner violence make it imperative that violence against women be more fully addressed during antenatal care.

Antenatal care can be a critical point of intervention for women suffering violence. Globally, 83 % of women have had at least one antenatal care visit [19], and even in low-resource settings women attending only one or a few antenatal care visits may benefit from an intervention which can be provided in a single session and can potentially reduce the occurrence of violence and mitigate its consequences. Other urgent health risks during pregnancy, such as HIV/AIDS, STI prevention, and malaria prophylaxis, are routinely addressed during antenatal care, however, identification and response to violence during pregnancy has not been routinely incorporated into antenatal care despite its potentially serious consequences and known adverse health outcomes. While the primary outcome of the study is to reduce partner violence, we also expect the intervention to have a positive impact on the mental health, safety and self-efficacy of participants.

Some health care providers understand how women's experiences of violence affect their health and the health of their infants, and many in South Africa and elsewhere have attempted to help their patients who are experiencing violence by a partner [20]. However, the evidence base is still limited for intervention strategies in the health sector and the antenatal care setting in particular. Two studies in high-income settings found significant positive results around a nurse-led antenatal intervention for IPV [21, 22]. Others found promising or insignificant results, although there was great variation in intensity and content of the intervention, and small sample sizes meant insufficient power to detect differences between groups [23].

While little evidence exists on effective interventions to identify and address violence in the health sector in low-resource settings, evidence from higher resource settings has shown some elements of promising interventions that could be adapted and replicated in other settings [24]. Empathetic listening is one of the most important elements of effective interventions to address violence. This, combined with discussions about safety planning and options for ending violence can be a valuable and empowering means of problem-solving and building self-efficacy in this context [21–23, 25, 26]. Some studies have found that having a focal person at the health centre who is specially trained on violence and dedicated to the issue can be an important element in responding to violence [27]; others identified support groups as a potentially positive intervention option [28]. In general, research has confirmed the value of interventions to address partner violence in antenatal care settings and suggests that even a single counselling session can result in improved safety behaviours and reduced levels of intimate partner violence [21, 22].

South Africa is an ideal setting to test such an intervention because of the high rates of antenatal care attendance. More than 92 % of women have at least one antenatal care visit and deliver with a skilled attendant [29]. In addition, South Africa has a high burden of HIV [30] and women with HIV are primarily identified in antenatal care. Violence against women and gender inequality are closely linked with HIV risk through several mechanisms. Violence or the threat of violence can limit HIV prevention behaviours, and disclosure of HIV status can provoke partner violence [31]. Therefore, it is critical that interventions to address violence against women in antenatal care are linked with HIV counselling and testing in South Africa as this study does.

## Methods/Design

A randomized controlled trial (RCT) of a two-session empowerment counselling intervention among pregnant women (*n* = 504) experiencing past-year physical and/or sexual IPV will be carried out at three antenatal clinics in Johannesburg, South Africa. Individual women will be randomized to the intervention or control group, with women in the intervention group receiving the empowerment counselling intervention and women in the control group receiving enhanced standard of care in the form of a list of referrals to local resources.

### Objectives

The project aims to achieve the following objectives:

1. To test a nurse-led counselling intervention for abused pregnant women receiving antenatal care in South Africa that could be incorporated into the routine antenatal care package.
2. To determine whether the counselling intervention being tested during pregnancy can reduce the recurrence of intimate partner violence and the frequency and severity of this violence.
3. To determine whether the counselling intervention being tested is effective in improving safety, empowerment, mental health, and help- and health-seeking behaviour of abused pregnant women.
4. To assess whether, at baseline, women experiencing intimate partner violence differ from women who do not experience intimate partner violence in regard to mental health, self-efficacy, and HIV risk behaviours.
5. To assess whether HIV-positive women who have experienced intimate partner violence differ from HIV-positive women who have not experienced intimate partner violence with regard to PMTCT uptake and adherence as measured at follow up.

The design of the randomized controlled trial was adapted following a formative research stage described elsewhere [32, 33], which included an assessment of the current response to violence against women in both the public and private sectors. Findings from the formative research validated the importance of having an appropriate response to violence against women in the clinic setting, and informants (providers and pregnant women) were supportive of the proposed intervention research [33]. The formative stage included identification of the organizations that respond to the health and psychosocial needs of women experiencing violence; examination of the current initiatives to address violence against women; assessment of the technical capacity of health workers to address violence and the barriers to implementation of a health sector response at present.

To avoid disclosing the nature of the research and to emphasize the focus on keeping women “safe” during pregnancy and the postpartum period, the study was named “Safe & Sound.”

**Fig. 1:**
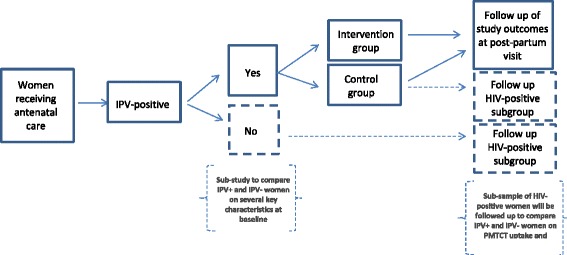
Study design provides a visual depiction of the study design

The randomized controlled trial (RCT) will test the efficacy of an intervention to address partner violence during pregnancy. Women screening positive for violence and who satisfy the eligibility criteria will be randomized to the intervention or control group (Fig. 1). The intervention group will receive up to two sessions of a counselling intervention and both groups will receive a brief information sheet with local referrals, including for counselling, shelters, legal resources, child protection services, police, and other relevant NGOs. Each of the listed referrals was contacted and its willingness and capacity to respond to referrals during the course of this study was assessed. Selected agencies will be included as active referral options. The protocol complies with the guidelines of the Standard Protocol Items: Recommendations for Interventional Trials (SPIRIT), and all data collection is in compliance with Consolidated Standards of Reporting Trials (CONSORT) guidelines.

At enrolment, data will be collected on socio-demographic characteristics of participants, including age, number of children, relationship and employment status, education level, housing type, length of stay in Johannesburg, socio-economic status and hunger index, country of origin, as well as number of pregnancies and live births for descriptive purposes and for potential inclusion in adjusted analyses.

### Primary outcome

Intimate partner violence will be measured using a modified version of the WHO instrument from the WHO Multi-Country Study on Women’s Health and Domestic Violence against Women [34]. This instrument assesses women’s experience of physical, sexual, and emotional violence as well as controlling behaviours by a male intimate partner. The effect of the intervention on the primary outcome of physical and/or sexual intimate partner violence will be assessed by comparing the percentage of women in the intervention vs. control groups reporting acts of physical and/or sexual partner violence during the study period and by analysing the patterns of frequency and intensity of the violence between the two study groups based on follow up data.

### Secondary outcomes

The two study arms will be compared on a number of secondary outcomes measured at follow-up, including mental health, safety planning, community resource use, and self-efficacy. The Hospital Anxiety and Depression Scale (HADS), a 21 question standardized instrument measuring anxiety and depression, will be used to assess the effect of the intervention on these mental health outcomes [35]. A score of 8 or above is considered clinically significant. An adapted version of the Safety Behavior Checklist [36] will be used to calculate an average score of safety behaviours used in the event of escalating violence. These include actions such as keeping an identity card available, keeping a bag packed in case of escalating violence, and removing knives or weapons from the home. Self-efficacy will be measured using the General Self-Efficacy Scale [37] and includes a set of questions on beliefs and actions indicating feelings of personal control over one’s life. Uptake of community resources, particularly those included on the study referral sheet, will also be assessed as a secondary study outcome by comparing scores between study arms.

### Sub-study outcomes

Two sub-studies will be linked to the RCT. One will assess the role of IPV on the uptake and adherence to prevention of mother-to-child transmission (PMTCT) among HIV-positive women. This sub-study will compare HIV-related outcomes in two groups of HIV-positive women: IPV-positive women in the control group and IPV-negative women who will be followed up for the purposes of this sub-study. Adherence to antiretroviral therapy among HIV-positive participants will be measured using the 30-day Visual Analog Scale (VAS). The VAS has been validated in South Africa [38], and ‘good adherence’ will be analysed using a cut-off of >95 % adherence. To further assess PMTCT, self-report questions will be adapted from a recent study among pregnant women in Cape Town, South Africa [39]. Each step in the PMTCT cascade will be analysed as a dichotomous variable (completed step vs. not completed).

A second sub-study will compare at baseline socio-demographic characteristics and mental health status of women experiencing intimate partner violence to those not experiencing partner violence to assess the role of IPV status on women’s mental health prior to enrolment in the study. These baseline measures will include post-traumatic stress (using the Harvard Trauma Questionnaire (HTQ) [40], anxiety and depression status, and selected socio-demographic characteristics. In addition, the association between IPV and selected socio-demographic characteristics will be assessed to determine which characteristics are associated with IPV independent of the intervention.

### Recruitment and randomization

Pregnant women will be screened for eligibility. Those who are at least 18 years old and less than 33 weeks gestation (to allow enough time for the second intervention session) and who are able to communicate in one of the most common local languages (English, Sotho, or Zulu) will be eligible to participate in the randomized controlled trial. Potential participants will also be asked if they have experienced any of a number of forms of partner violence in the past 12 months. Only those reporting that they have experienced physical or sexual violence by their current or most recent partner in the past 12 months will be eligible. Prior to being enrolled, women will also be screened for immediate safety risk. Reported risk of imminent lethal violence by a partner, fear that a child in the home is at immediate risk of lethal violence by the partner, and being at suicidal risk (as determined by having ideation with a plan to commit suicide) will result in an immediate referral and disqualify women from participating.

Women will be recruited during their routine antenatal appointments at primary healthcare clinics in urban Johannesburg. All clinics are part of the district public health service and generally serve very low income communities. The study will be introduced by a research nurse, who will provide a group introduction to the study without identifying the study as being related to violence but rather framing it as a study on women’s health during pregnancy. A study flier will be distributed with the name of the study and a phone number and first name of the research nurse assigned to that clinic site. Interested women will be offered an opportunity to speak to nurses following their routine clinic care or to schedule an appointment for another time.

Randomization will be conducted through block randomization procedures with study arm assignment revealed upon opening of sequential opaque sealed envelopes prepared in Geneva by the Department of Reproductive Health and Research, World Health Organization. Study nurses will not be blinded to study arm; the same nurse who conducts the baseline interview will also randomize eligible participants.

### Sample size

The sample size needed to detect a difference in the primary outcome (IPV reduction) between the intervention group and the control group will be 252 per group, based on sample size calculations using results from previous studies testing a similar intervention in which a difference of 7 % was detected between the intervention and control group on intimate partner violence experience following the intervention [23].^1^ Nurse researchers will be able to screen between one to five women per clinic session. Based on previous studies [4], we estimate that 25 % of women will report experiencing physical or sexual violence from their partner in the last 12 months. Nurse researchers will interview and randomize the eligible participants as well as those who are not eligible for the RCT but who are eligible for the sub-studies based on responses in the baseline questionnaire.

### Follow up

Women will be followed up either at their six weeks’ post-partum visit, when they come for their post-natal check-up or immunization for their baby, by phone or at a time arranged with the study nurse. An estimated 15 % of loss to follow up is expected based on other studies in this setting.

All women who experience an adverse event or serious adverse event will be followed up until it is resolved or she is in stable care. For the purposes of this study, an adverse event will be defined as any untoward medical occurrence or harmful event that affects the health or well-being of a study participant or her infant while participating in the study. A serious adverse event is defined as any untoward medical occurrence or harmful event that results in the death of a participant or her infant, is life-threatening, requires inpatient hospitalization (other than for routine delivery) or prolongation of existing hospitalization, or results in persistent or significant disability.

A recruitment period of 18 months with an additional 6 months of follow-up will be needed to recruit a sufficiently large sample size, assuming exclusions for ineligibility, refusal to participate, and loss to follow up.

### The intervention

The Safe & Sound intervention is based on a nurse-led "empowerment counselling model". This model has been tested through randomized controlled studies in antenatal care and shown to be effective in improving women's safety, coping, and potentially in reducing violence in the United States [21, 23] and Hong Kong [22]. The empowerment model assumes that the perpetration of intimate partner violence aims to control the behaviour of female partners and that by increasing a woman's sense of control over her life, she can better develop strategies to reduce violence in her relationship [41]. This model promoted by Dutton [26] includes two complementary components-- (a) improving women's safety and protection, including negotiating safer sex with a partner, while (b) enhancing her decision-making and problem solving ability in her relationship.

Safe & Sound is provided by a nurse researcher trained in counselling and covers a combination of the following elements that will be individually tailored depending on women’s experience and her readiness to change:

- **Empathetic listening;** also called active listening or reflective listening is a way of listening and responding to another person that improves mutual understanding and trust, including emotional identification, compassion, feeling, and insight.
- **Cyclical nature of partner violence:** the nurse researcher will discuss how abusive relationships often follow predictable patterns in which abuse might subside for a period of time, only to resume again later. She will also discuss possible warning signs that could indicate that a renewed phase of violence might be imminent. By better understanding these patterns, the woman should be better prepared to prevent or reduce subsequent violence and to predict signs of increased dang**e**r.
- **Evaluating danger and discussing options:** the nurse will help the woman assess her level of risk in her relationship with the violent partner and whether there are signs that the violence will escalate with potential health consequences for her and her pregnancy. If the woman or her children are in immediate danger for their life or if the woman discloses suicidal ideation, she will not be part of the study and will be referred appropriately, as discussed previously. Based on the woman's current risk and the support available to her, the nurse will discuss options for reducing abuse, without providing directives or value judgements. The nurse will recognize that women cannot always safely leave their abusive partners, but that they have options for reducing abuse in their relationship. These options include protecting children from violence, developing safety strategies, accessing community resources for social support, and addressing relationship dynamics with partners.
- **Developing safety strategies:** the nurse will help the woman to develop a safety strategy tailored to her situation and based on culturally appropriate actions for increasing personal safety of herself and her children. These strategies include developing a code with family and friends to indicate increased risk, alerting neighbours to the situation and seeking support from them in the case of an abusive incident, having a bag and documents ready in case of the need to flee with children in the event of imminent danger. Safety will also be discussed in relation to abused women’s increased vulnerability to HIV or inability to seek appropriate treatment among HIV positive women, especially PMTCT. The nurse researcher will help the woman identify those behaviours and strategies that she can best implement.
- **Pregnancy changes:** based on early piloting of the intervention which suggested women have concerns around relationship and sexuality changes in pregnancy, nurses can begin an open ended discussion around participants’ pregnancy-related concerns, including changes in relationship dynamics, financial needs, or sexual desires that couples might experience during the pregnancy and post-partum period. The approach is to listen empathetically and provide accurate health-related information to a number of concerns (such as perceived danger of sexual intercourse during pregnancy).
- **Legal steps:** women who are prepared to take legal steps to protect themselves or their children from a violent partner are given step-by-step instructions for obtaining a protection order. In practice, this process is complex and requires many visits to the magistrate court and police. A ‘job aid’ developed with input from a local legal organization specializing in IPV will be employed. If needed, nurses can refer women to legal aid for support in completing the protection order.
- **Available resources:** based on the findings of the formative research, participants will be given a list of organizations or social services available locally that can provide help with the psychological, legal, social, shelter, or health-related needs resulting from violence. All agencies will have been sensitized to the study and have demonstrated capacity to serve as referrals. The nurses will have a choice of giving women a written, verbal, or telephone referral to the external organizations, depending on the preferences of the woman.

### Training

The 30-h technical training of nurse researchers will be carried out over a one-week period and will be based on a training manual developed for the study, which will include a combination of presentations, participatory activities, readings, reflections, and role-play exercises. The following topics will be covered in the manual and training curriculum: improving knowledge and awareness around intimate partner violence and how it links with maternal and child health issues, and specifically with HIV transmission and adherence to PMTCT; understanding the legal rights of women experiencing violence in the study context; promoting mental health among the study population; awareness of previous and current trauma in the lives of participants; child protection services and reporting obligations within the legal framework; cycle of violence patterns; behaviour change and assessing women’s readiness to change; safety planning; community resources; self-care, relaxation, and dealing with vicarious trauma. Clarifying values and perceptions about violence based on individual or observed experiences is also an important component of the training. A trained clinical psychologist and social workers will be available for counselling and discussion with the nurse researchers in the event they feel the need for emotional support or if they experience an emotional response to the content of the training. The nurse researchers will also be trained on the ethical and safety issues specific to research on violence against women and how to identify and report on adverse events and serious adverse events.

Weekly team meetings will offer a chance to debrief about patient encounters and discuss relevant “case studies”. This process will further familiarize nurses with the components of the intervention and how to implement a second session. Throughout the discussion of “case studies”, the study investigators will provide feedback to the team on how to implement the components of the intervention in a standardized yet tailored manner. Weekly meetings will also be an opportunity to stress the importance of ensuring that participants randomized to the control group are not intentionally or inadvertently given components of the intervention, which is a potential limitation in the study design. Group supervision will be supplemented by a trained social worker affiliated with the study, who can give guidance to nurse researchers on patient care as needed.

### Process evaluation

Process evaluations of clinical trials are important for understanding and interpreting findings from a trial, in particular why an intervention may or may not lead to intended outcomes [42, 43]. A process evaluation will be conducted over the course of the study to evaluate the feasibility and safety of the intervention implementation, the strengths and weaknesses of the intervention content, and the mechanisms through which the intervention impacts outcomes. For this component, a mixed methods approach will be used. Qualitative methods will include in-depth interviews with participants, nurse researchers and health workers in the study clinics; observations of the intervention sessions; structured reflection diaries kept by nurse researchers; and review of meeting reports from team meetings with nurse researchers discussing study implementation. Quantitative methods will include review of safety information from case report forms, review of adverse event and serious adverse event reports, analysis of intervention checklist reporting form completed by nurses for each participant, analysis of safety related questions in the database. The process evaluation will explore the factors that helped or hindered women’s uptake and use of the intervention, factors that helped the nurses provide the intervention effectively, as well as improving understanding about the pathways of change for women who responded to the intervention. The practical considerations of implementing a health sector based intervention will be assessed, and the potential mechanisms of how the intervention impacts change will also be explored.

### Data management

Data collection will be done on paper case report forms (CRFs) and data kept in locked cabinets until they are transferred to the central office and reviewed in a data quality check by the data manager. The data manager will also compare responses in the screening logs with responses in the CRFs to confirm the accuracy of the screening logs. Since patient records are not kept at clinics but rather are carried with them from appointment to appointment, the screening logs and some components of the CRFs will be considered source documents for the trial. Screening logs and CRFs will be scanned and uploaded to a secure location accessible by the Geneva data management team. Data entry will occur at WHO in Geneva. Open Clinica software [44] will be used for data management.

The UNDP-UNFPA-UNICEF-WHO-World Bank Special Programme of Research, Development and Research Training in Human Reproduction (HRP) serves as sponsor for the study. Clinical studies supported by WHO are carried out according to the International Conference on Harmonization (ICH) Good Clinical Practice (GCP) standards, and WHO/HRP standard operating procedures (WHO/HRP SOPs) in the design of the study, data collection, management, analysis, and interpretation of data. HRP standard operating procedures (SOPs) for managing clinical trials will be adhered to throughout the trial. These procedures have been used in previous trials sponsored or coordinated by WHO. The roles and responsibilities of all individuals and institutions involved in the design and conduct of the study, including the study sponsor, study staff, study investigators, steering committee, and the Data Safety and Monitoring Board (DSMB) are clearly described in the SOPs. Data quality will be monitored through 100 % visual checking of CRFs and screening logs at site by data manager and through additional visual checks by the data analyst in Geneva. There will also be query reporting once data are entered into the data management system and through file reviews during regular monitoring visits.

Data will be analysed by study arm using an intention to treat analysis and as detailed in a data analysis plan developed for the study. Statistical data will be analysed using Stata [45] and qualitative data from the process evaluation will be analysed using NVivo [46].

### Ethical and safety considerations

Researching violence against women can potentially result in unintentional risks to participants, researchers, and other health personnel involved in the research. To reduce risks, the study will follow strict ethical and safety guidelines, based on the WHO recommendations in *Putting women first: ethical and safety recommendations for research on domestic violence against women* [47]. These include standard ethical measures on informed consent, confidentiality, and voluntary participation, as well as additional measures related to risks in conducting research on violence against women. For example, nurse researchers are specially trained in inquiring about violence experience sensitively and in a supportive manner. They are expected to carry out all interviews in total privacy and keep all information, in particular intimate partner violence and HIV status of women, confidential. They will also invite women to keep their involvement in the study to themselves, to avoid any safety risk from study participation. Any mention of the study by the study team or clinic nurses will describe the study as a study on women’s health and safety during pregnancy. This type of language prevents stigmatization of women participating in the study. Referral networks have been established so that women can seek additional services from organizations in the community.

All adverse events and serious adverse events reported by participants or by clinic staff to study nurses will be documented and reviewed by study investigators to determine whether the event was unrelated or possibly, probably or highly probably related to IPV and to study participation. All events will be followed up until resolved and medical records will be sought when further information is required. Those events deemed possibly, probably or highly probably related to study participation will be escalated and reported to the Data Safety and Monitoring Board (DSMB). The DSMB will be comprised of a statistician, an epidemiologist and a researcher specializing in gender-based violence and will conduct periodic reviews of the data when 25 %, 50 %, 75 % and 100 % of the blinded follow up data of the target sample is collected. They can request the unblinding of the data and the discontinuation of the study if warranted by the preliminary results. They will review monthly reports of adverse events and serious adverse events and will review all adverse events or serious adverse events assessed by investigators as being”probably” or “highly probably” related to study participation. The DSMB will advise on any protective actions that should be taken.

Ethical clearance was granted by the Human Research Ethics Committee of the University of the Witwatersrand, Johannesburg, South Africa (M110832) and the Ethical Review Committee of the World Health Organization in Geneva, Switzerland (RPC471).

## Discussion

The results of this study will provide much needed evidence on how to address the needs of abused women in an antenatal care package and on how to implement an appropriate health sector response to violence against women in low-resource settings. Testing an intervention under controlled conditions to determine efficacy is an important step prior to integrating it into a clinic setting and determining how to implement it effectively. This study is designed to test the efficacy of an intervention and as such, dedicated research nurses will be hired and trained to carry out the research aspects (e.g., determining eligibility, collecting data, randomizing eligible participants) as well as the clinical aspects of implementing the intervention or control conditions.

Several limitations should be noted. The overlapping roles of research nurses in managing the research and intervention aspects and the lack of blinding of study nurses are important limitations in the study design. Since nurses are trained in how to implement the intervention it will potentially be difficult or professionally distressing for them to maintain a control condition throughout the study. This will be addressed thoroughly in the training and team meetings.

It was decided that for ethical reasons the control group would receive enhanced standard of care, a referral services list, rather than receiving standard level of care, which in South Africa is presently nothing at all. Women in the study may benefit from the referral list as well as from the baseline encounter with the nurse in which she inquires about her experiences of violence in a kind, non-judgmental way. Inquiring about violence exposure in the context of research may lead women to contemplate the role of violence in their lives, which could increase their awareness and recognition of the problem and potentially influence outcomes [48]. These conditions may reduce the relative difference in outcomes between the intervention and control groups and could bias results towards the null hypothesis. These limitations will be monitored during the study through the process evaluation and regular study monitoring to understand their influence on the study outcomes.

Despite these limitations, the study will add important evidence on the efficacy of a health sector intervention to reduce partner violence in a context that urgently requires practical intervention. As was confirmed in the findings of the formative research stage, integrating this type of intervention into antenatal care settings, particularly with HIV- counselling and testing components, would be an appropriate response in this setting. The providers and patients interviewed confirmed that such an intervention would be well received and could be an important step in supporting women during pregnancy to improve their health and that of their children.

## Abbreviations

DSMB: Data safety and monitoring board
HADS: Hospital anxiety and depression scale
HIV: Human immunodeficiency virus
HTQ: Harvard trauma questionnaire
IPV: Intimate partner violence
PMTCT: Prevention of mother-to-child transmission
RCT: Randomized controlled trial
VAS: Visual analog scale.

## Footnotes

1 Sample size calculations assume significance level = .05 and power = 80 %.
